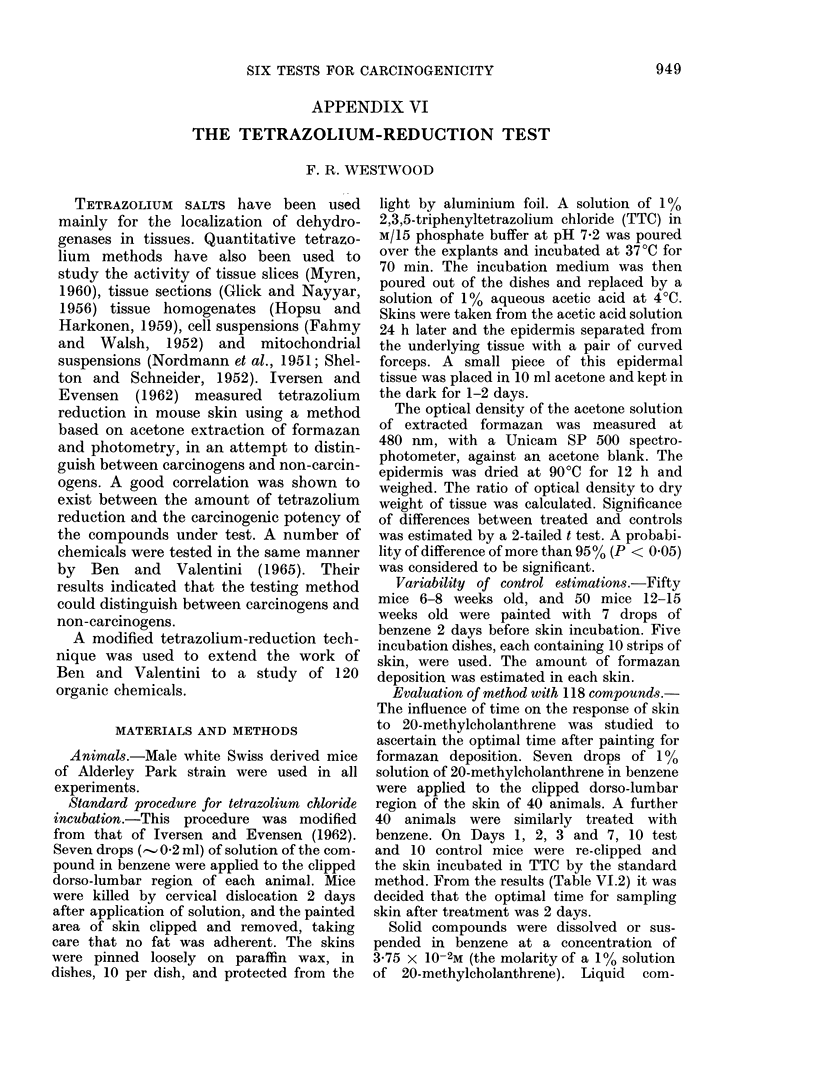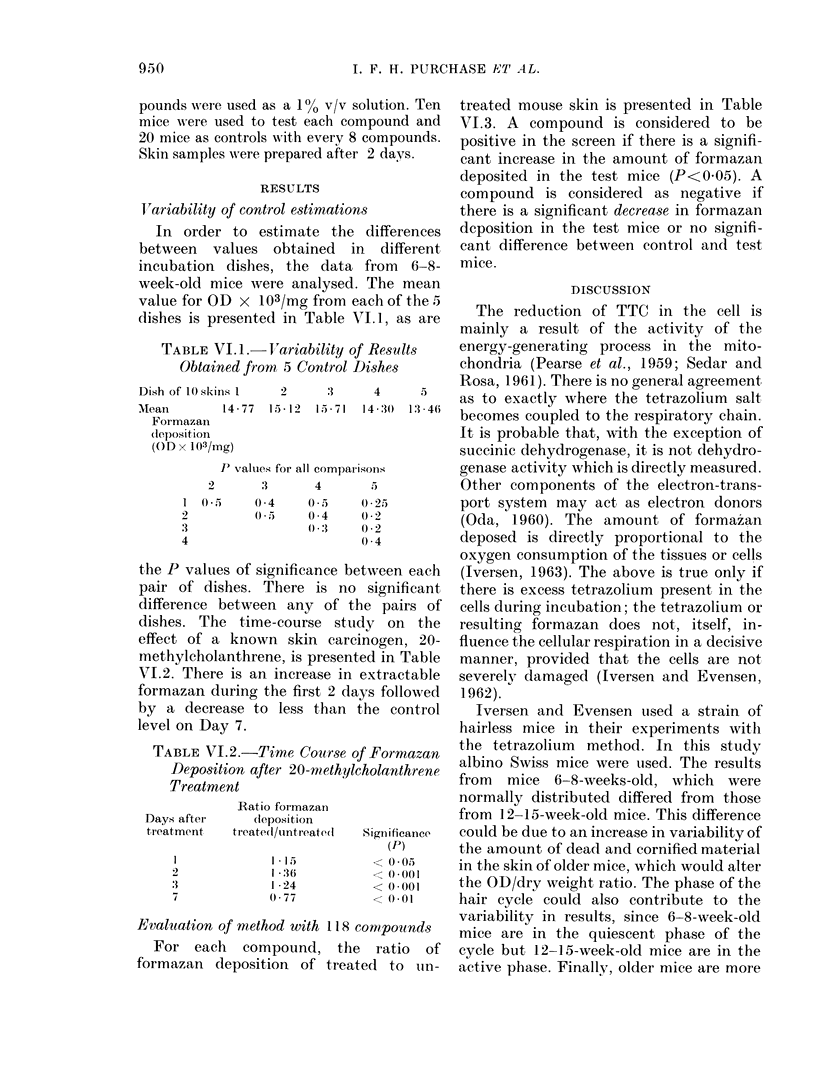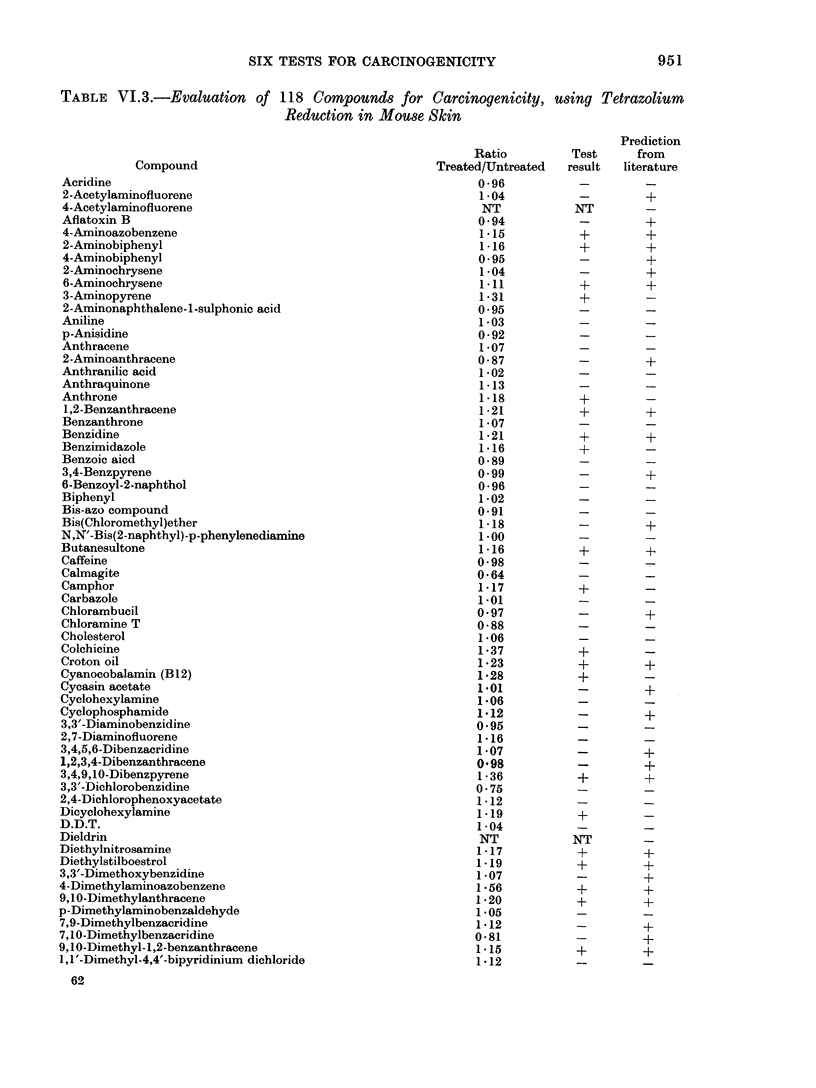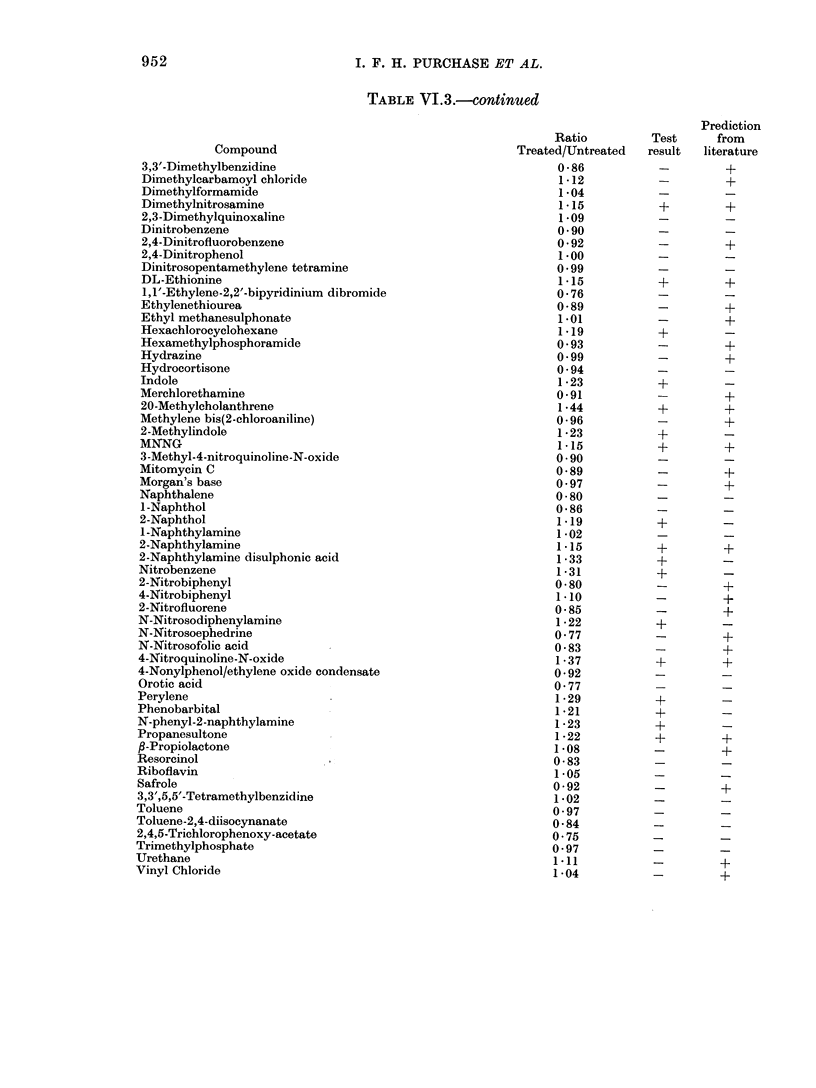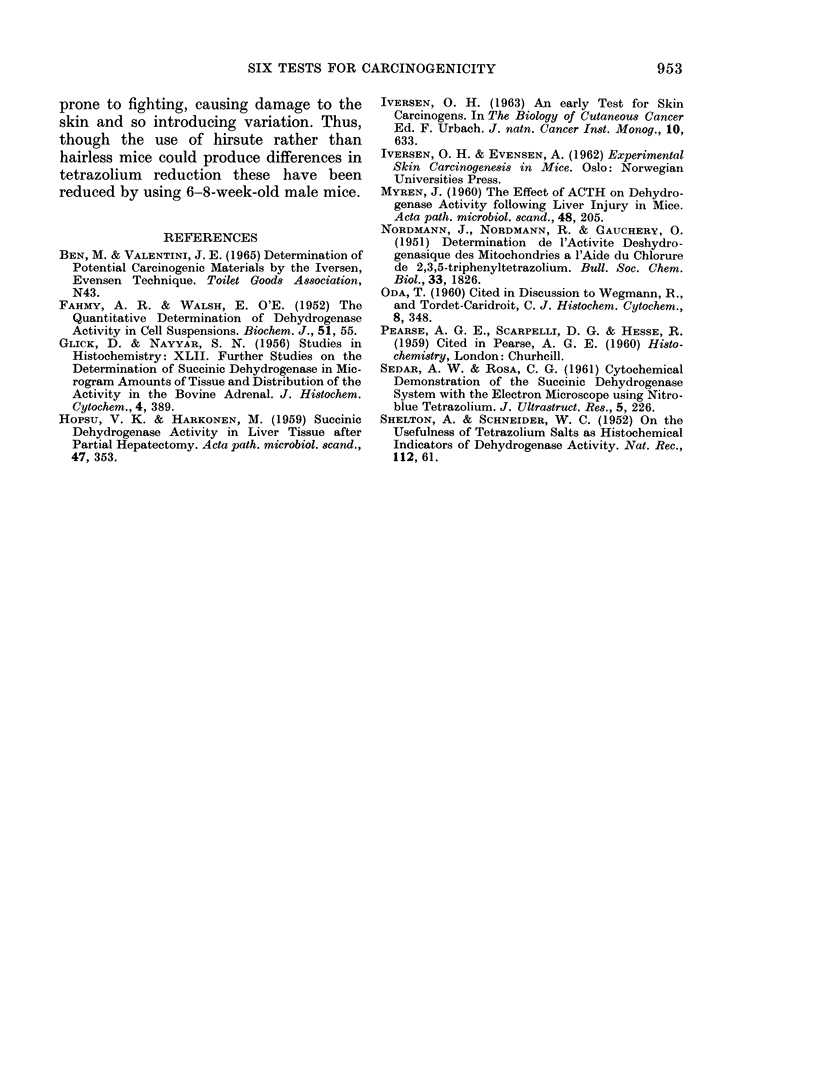# The tetrazolium-reduction test. Six tests for carcinogenicity.

**DOI:** 10.1038/bjc.1978.138

**Published:** 1978-06

**Authors:** F. R. Westwood


### APPENDIX VI

### THE TETRAZOLIUM-REDUCTION TEST

F. R. WESTWOOD

TETRAZOLIUM SALTS have been used mainly for the localization of dehydrogenases in tissues. Quantitative tetrazolium methods have also been used to study the activity of tissue slices (Myren, 1960), tissue sections (Glick and Nayyar, 1956) tissue homogenates (Hopsu and Harkonen, 1959), cell suspensions (Fahmy and Walsh, 1952) and mitochondrial suspensions (Nordmann *et al.*, 1951; Shelton and Schneider, 1952). Iversen and Evensen (1962) measured tetrazolium reduction in mouse skin using a method based on acetone extraction of formazan and photometry, in an attempt to distinguish between carcinogens and non-carcinogens. A good correlation was shown to exist between the amount of tetrazolium reduction and the carcinogenic potency of the compounds under test. A number of chemicals were tested in the same manner by Ben and Valentini (1965). Their results indicated that the testing method could distinguish between carcinogens and non-carcinogens.

A modified tetrazolium-reduction technique was used to extend the work of Ben and Valentini to a study of 120 organic chemicals.

### MATERIALS AND METHODS

*Animals.*—Male white Swiss derived mice of Alderley Park strain were used in all experiments.

*Standard procedure for tetrazolium chloride incubation.*—This procedure was modified from that of Iversen and Evensen (1962). Seven drops ($\sim$0·2 ml) of solution of the compound in benzene were applied to the clipped dorso-lumbar region of each animal. Mice were killed by cervical dislocation 2 days after application of solution, and the painted area of skin clipped and removed, taking care that no fat was adherent. The skins were pinned loosely on paraffin wax, in dishes, 10 per dish, and protected from the light by aluminium foil. A solution of 1% 2,3,5-triphenyltetrazolium chloride (TTC) in M/15 phosphate buffer at pH 7·2 was poured over the explants and incubated at 37°C for 70 min. The incubation medium was then poured out of the dishes and replaced by a solution of 1% aqueous acetic acid at 4°C. Skins were taken from the acetic acid solution 24 h later and the epidermis separated from the underlying tissue with a pair of curved forceps. A small piece of this epidermal tissue was placed in 10 ml acetone and kept in the dark for 1–2 days.

The optical density of the acetone solution of extracted formazan was measured at 480 nm, with a Unicam SP 500 spectrophotometer, against an acetone blank. The epidermis was dried at 90°C for 12 h and weighed. The ratio of optical density to dry weight of tissue was calculated. Significance of differences between treated and controls was estimated by a 2-tailed $t$ test. A probability of difference of more than 95% ($P < 0{\cdot}05$) was considered to be significant.

*Variability of control estimations.*—Fifty mice 6–8 weeks old, and 50 mice 12–15 weeks old were painted with 7 drops of benzene 2 days before skin incubation. Five incubation dishes, each containing 10 strips of skin, were used. The amount of formazan deposition was estimated in each skin.

*Evaluation of method with* 118 *compounds.*—The influence of time on the response of skin to 20-methylcholanthrene was studied to ascertain the optimal time after painting for formazan deposition. Seven drops of 1% solution of 20-methylcholanthrene in benzene were applied to the clipped dorso-lumbar region of the skin of 40 animals. A further 40 animals were similarly treated with benzene. On Days 1, 2, 3 and 7, 10 test and 10 control mice were re-clipped and the skin incubated in TTC by the standard method. From the results (Table VI.2) it was decided that the optimal time for sampling skin after treatment was 2 days.

Solid compounds were dissolved or suspended in benzene at a concentration of $3{\cdot}75 \times 10^{-2}$M (the molarity of a 1% solution of 20-methylcholanthrene). Liquid compounds were used as a 1% v/v solution. Ten mice were used to test each compound and 20 mice as controls with every 8 compounds. Skin samples were prepared after 2 days.

### RESULTS

### *Variability of control estimations*

In order to estimate the differences between values obtained in different incubation dishes, the data from 6–8-week-old mice were analysed. The mean value for OD $\times$ $10^3$/mg from each of the 5 dishes is presented in Table VI.1, as are the $P$ values of significance between each pair of dishes. There is no significant difference between any of the pairs of dishes. The time-course study on the effect of a known skin carcinogen, 20-methylcholanthrene, is presented in Table VI.2. There is an increase in extractable formazan during the first 2 days followed by a decrease to less than the control level on Day 7.

TABLE VI.1.—*Variability of Results Obtained from 5 Control Dishes*

| Dish of 10 skins | 1 | 2 | 3 | 4 | 5 |
|---|---|---|---|---|---|
| Mean Formazan deposition (OD $\times$ $10^3$/mg) | 14·77 | 15·12 | 15·71 | 14·30 | 13·46 |

*P* values for all comparisons

| | 2 | 3 | 4 | 5 |
|---|---|---|---|---|
| 1 | 0·5 | 0·4 | 0·5 | 0·25 |
| 2 | | 0·5 | 0·4 | 0·2 |
| 3 | | | 0·3 | 0·2 |
| 4 | | | | 0·4 |

TABLE VI.2.—*Time Course of Formazan Deposition after* 20-*methylcholanthrene Treatment*

| Days after treatment | Ratio formazan deposition treated/untreated | Significance ($P$) |
|---|---|---|
| 1 | 1·15 | $<$ 0·05 |
| 2 | 1·36 | $<$ 0·001 |
| 3 | 1·24 | $<$ 0·001 |
| 7 | 0·77 | $<$ 0·01 |

### *Evaluation of method with* 118 *compounds*

For each compound, the ratio of formazan deposition of treated to untreated mouse skin is presented in Table VI.3. A compound is considered to be positive in the screen if there is a significant increase in the amount of formazan deposited in the test mice ($P<0·05$). A compound is considered as negative if there is a significant *decrease* in formazan deposition in the test mice or no significant difference between control and test mice.

### DISCUSSION

The reduction of TTC in the cell is mainly a result of the activity of the energy-generating process in the mitochondria (Pearse *et al.*, 1959; Sedar and Rosa, 1961). There is no general agreement as to exactly where the tetrazolium salt becomes coupled to the respiratory chain. It is probable that, with the exception of succinic dehydrogenase, it is not dehydrogenase activity which is directly measured. Other components of the electron-transport system may act as electron donors (Oda, 1960). The amount of formazan deposed is directly proportional to the oxygen consumption of the tissues or cells (Iversen, 1963). The above is true only if there is excess tetrazolium present in the cells during incubation; the tetrazolium or resulting formazan does not, itself, influence the cellular respiration in a decisive manner, provided that the cells are not severely damaged (Iversen and Evensen, 1962).

Iversen and Evensen used a strain of hairless mice in their experiments with the tetrazolium method. In this study albino Swiss mice were used. The results from mice 6–8-weeks-old, which were normally distributed differed from those from 12–15-week-old mice. This difference could be due to an increase in variability of the amount of dead and cornified material in the skin of older mice, which would alter the OD/dry weight ratio. The phase of the hair cycle could also contribute to the variability in results, since 6–8-week-old mice are in the quiescent phase of the cycle but 12–15-week-old mice are in the active phase. Finally, older mice are more

TABLE VI.3.—*Evaluation of* 118 *Compounds for Carcinogenicity, using Tetrazolium Reduction in Mouse Skin*

| Compound | Ratio Treated/Untreated | Test result | Prediction from literature |
|---|---|---|---|
| Acridine | 0·96 | – | – |
| 2-Acetylaminofluorene | 1·04 | – | + |
| 4-Acetylaminofluorene | NT | NT | – |
| Aflatoxin B | 0·94 | – | + |
| 4-Aminoazobenzene | 1·15 | + | + |
| 2-Aminobiphenyl | 1·16 | + | + |
| 4-Aminobiphenyl | 0·95 | – | + |
| 2-Aminochrysene | 1·04 | – | + |
| 6-Aminochrysene | 1·11 | + | + |
| 3-Aminopyrene | 1·31 | + | – |
| 2-Aminonaphthalene-1-sulphonic acid | 0·95 | – | – |
| Aniline | 1·03 | – | – |
| p-Anisidine | 0·92 | – | – |
| Anthracene | 1·07 | – | – |
| 2-Aminoanthracene | 0·87 | – | + |
| Anthranilic acid | 1·02 | – | – |
| Anthraquinone | 1·13 | – | – |
| Anthrone | 1·18 | + | – |
| 1,2-Benzanthracene | 1·21 | + | + |
| Benzanthrone | 1·07 | – | – |
| Benzidine | 1·21 | + | + |
| Benzimidazole | 1·16 | + | – |
| Benzoic aicd | 0·89 | – | – |
| 3,4-Benzpyrene | 0·99 | – | + |
| 6-Benzoyl-2-naphthol | 0·96 | – | – |
| Biphenyl | 1·02 | – | – |
| Bis-azo compound | 0·91 | – | – |
| Bis(Chloromethyl)ether | 1·18 | – | + |
| N,N′-Bis(2-naphthyl)-p-phenylenediamine | 1·00 | – | – |
| Butanesultone | 1·16 | + | + |
| Caffeine | 0·98 | – | – |
| Calmagite | 0·64 | – | – |
| Camphor | 1·17 | + | – |
| Carbazole | 1·01 | – | – |
| Chlorambucil | 0·97 | – | + |
| Chloramine T | 0·88 | – | – |
| Cholesterol | 1·06 | – | – |
| Colchicine | 1·37 | + | – |
| Croton oil | 1·23 | + | + |
| Cyanocobalamin (B12) | 1·28 | + | – |
| Cycasin acetate | 1·01 | – | + |
| Cyclohexylamine | 1·06 | – | – |
| Cyclophosphamide | 1·12 | – | + |
| 3,3′-Diaminobenzidine | 0·95 | – | – |
| 2,7-Diaminofluorene | 1·16 | – | – |
| 3,4,5,6-Dibenzacridine | 1·07 | – | + |
| 1,2,3,4-Dibenzanthracene | 0·98 | – | + |
| 3,4,9,10-Dibenzpyrene | 1·36 | + | + |
| 3,3′-Dichlorobenzidine | 0·75 | – | – |
| 2,4-Dichlorophenoxyacetate | 1·12 | – | – |
| Dicyclohexylamine | 1·19 | + | – |
| D.D.T. | 1·04 | – | – |
| Dieldrin | NT | NT | – |
| Diethylnitrosamine | 1·17 | + | + |
| Diethylstilboestrol | 1·19 | + | + |
| 3,3′-Dimethoxybenzidine | 1·07 | – | + |
| 4-Dimethylaminoazobenzene | 1·56 | + | + |
| 9,10-Dimethylanthracene | 1·20 | + | + |
| p-Dimethylaminobenzaldehyde | 1·05 | – | – |
| 7,9-Dimethylbenzacridine | 1·12 | – | + |
| 7,10-Dimethylbenzacridine | 0·81 | – | + |
| 9,10-Dimethyl-1,2-benzanthracene | 1·15 | + | + |
| 1,1′-Dimethyl-4,4′-bipyridinium dichloride | 1·12 | – | – |

TABLE VI.3.—*continued*

| Compound | Ratio Treated/Untreated | Test result | Prediction from literature |
|---|---|---|---|
| 3,3′-Dimethylbenzidine | 0·86 | − | + |
| Dimethylcarbamoyl chloride | 1·12 | − | + |
| Dimethylformamide | 1·04 | − | − |
| Dimethylnitrosamine | 1·15 | + | + |
| 2,3-Dimethylquinoxaline | 1·09 | − | − |
| Dinitrobenzene | 0·90 | − | − |
| 2,4-Dinitrofluorobenzene | 0·92 | − | + |
| 2,4-Dinitrophenol | 1·00 | − | − |
| Dinitrosopentamethylene tetramine | 0·99 | − | − |
| DL-Ethionine | 1·15 | + | + |
| 1,1′-Ethylene-2,2′-bipyridinium dibromide | 0·76 | − | − |
| Ethylenethiourea | 0·89 | − | + |
| Ethyl methanesulphonate | 1·01 | − | + |
| Hexachlorocyclohexane | 1·19 | + | − |
| Hexamethylphosphoramide | 0·93 | − | + |
| Hydrazine | 0·99 | − | + |
| Hydrocortisone | 0·94 | − | − |
| Indole | 1·23 | + | − |
| Merchlorethamine | 0·91 | − | + |
| 20-Methylcholanthrene | 1·44 | + | + |
| Methylene bis(2-chloroaniline) | 0·96 | − | + |
| 2-Methylindole | 1·23 | + | − |
| MNNG | 1·15 | + | + |
| 3-Methyl-4-nitroquinoline-N-oxide | 0·90 | − | − |
| Mitomycin C | 0·89 | − | + |
| Morgan's base | 0·97 | − | + |
| Naphthalene | 0·80 | − | − |
| 1-Naphthol | 0·86 | − | − |
| 2-Naphthol | 1·19 | + | − |
| 1-Naphthylamine | 1·02 | − | − |
| 2-Naphthylamine | 1·15 | + | + |
| 2-Naphthylamine disulphonic acid | 1·33 | + | − |
| Nitrobenzene | 1·31 | + | − |
| 2-Nitrobiphenyl | 0·80 | − | + |
| 4-Nitrobiphenyl | 1·10 | − | + |
| 2-Nitrofluorene | 0·85 | − | + |
| N-Nitrosodiphenylamine | 1·22 | + | − |
| N-Nitrosoephedrine | 0·77 | − | + |
| N-Nitrosofolic acid | 0·83 | − | + |
| 4-Nitroquinoline-N-oxide | 1·37 | + | + |
| 4-Nonylphenol/ethylene oxide condensate | 0·92 | − | − |
| Orotic acid | 0·77 | − | − |
| Perylene | 1·29 | + | − |
| Phenobarbital | 1·21 | + | − |
| N-phenyl-2-naphthylamine | 1·23 | + | − |
| Propanesultone | 1·22 | + | + |
| β-Propiolactone | 1·08 | − | + |
| Resorcinol | 0·83 | − | − |
| Riboflavin | 1·05 | − | − |
| Safrole | 0·92 | − | + |
| 3,3′,5,5′-Tetramethylbenzidine | 1·02 | − | − |
| Toluene | 0·97 | − | − |
| Toluene-2,4-diisocynanate | 0·84 | − | − |
| 2,4,5-Trichlorophenoxy-acetate | 0·75 | − | − |
| Trimethylphosphate | 0·97 | − | − |
| Urethane | 1·11 | − | + |
| Vinyl Chloride | 1·04 | − | + |

prone to fighting, causing damage to the skin and so introducing variation. Thus, though the use of hirsute rather than hairless mice could produce differences in tetrazolium reduction these have been reduced by using 6–8-week-old male mice.

### REFERENCES

Ben, M. & Valentini, J. E. (1965) Determination of Potential Carcinogenic Materials by the Iversen, Evensen Technique. *Toilet Goods Association*, N43.

Fahmy, A. R. & Walsh, E. O'E. (1952) The Quantitative Determination of Dehydrogenase Activity in Cell Suspensions. *Biochem. J.*, **51**, 55.

Glick, D. & Nayyar, S. N. (1956) Studies in Histochemistry: XLII. Further Studies on the Determination of Succinic Dehydrogenase in Microgram Amounts of Tissue and Distribution of the Activity in the Bovine Adrenal. *J. Histochem. Cytochem.*, **4**, 389.

Hopsu, V. K. & Harkonen, M. (1959) Succinic Dehydrogenase Activity in Liver Tissue after Partial Hepatectomy. *Acta path. microbiol. scand.*, **47**, 353.

Iversen, O. H. (1963) An early Test for Skin Carcinogens. In *The Biology of Cutaneous Cancer* Ed. F. Urbach. *J. natn. Cancer Inst. Monog.*, **10**, 633.

Iversen, O. H. & Evensen, A. (1962) *Experimental Skin Carcinogenesis in Mice*. Oslo: Norwegian Universities Press.

Myren, J. (1960) The Effect of ACTH on Dehydrogenase Activity following Liver Injury in Mice. *Acta path. microbiol. scand.*, **48**, 205.

Nordmann, J., Nordmann, R. & Gauchery, O. (1951) Determination de l'Activite Deshydrogenasique des Mitochondries a l'Aide du Chlorure de 2,3,5-triphenyltetrazolium. *Bull. Soc. Chem. Biol.*, **33**, 1826.

Oda, T. (1960) Cited in Discussion to Wegmann, R., and Tordet-Caridroit, C. *J. Histochem. Cytochem.*, **8**, 348.

Pearse, A. G. E., Scarpelli, D. G. & Hesse, R. (1959) Cited in Pearse, A. G. E. (1960) *Histochemistry*, London: Churhcill.

Sedar, A. W. & Rosa, C. G. (1961) Cytochemical Demonstration of the Succinic Dehydrogenase System with the Electron Microscope using Nitroblue Tetrazolium. *J. Ultrastruct. Res.*, **5**, 226.

Shelton, A. & Schneider, W. C. (1952) On the Usefulness of Tetrazolium Salts as Histochemical Indicators of Dehydrogenase Activity. *Nat. Rec.*, **112**, 61.